# Lip Melanosis Postdermal Filler Injection: Second Case Report and Literature Review

**DOI:** 10.1155/crid/4265486

**Published:** 2025-01-07

**Authors:** Abdulrahman Abdullah Altariqi, Azizah Fahad Bin Mubayrik

**Affiliations:** ^1^College of Dentistry, King Saud University, Riyadh, Saudi Arabia; ^2^Division of Oral Medicine, Department of Oral Medicine and Diagnostic Sciences, College of Dentistry, King Saud University, Riyadh, Saudi Arabia

**Keywords:** complications, filler-related, lip, melanosis

## Abstract

Oral pigmentation can arise from various factors, including physiological and pathological, or as a manifestation of an underlying systemic disease. We present an atypical case of dermal filler–related complication, in which clinical lip pigmentation was observed. This condition can pose a diagnostic challenge in accurately identifying its cause.

## 1. Introduction

The oral mucosa or gingiva may become discolored due to various diseases and disorders. This discoloration is called pigmentation [1]. Pigmentations in the oral cavity typically arise from either external or internal factors (Table 1) [2]. External pigmentation, for instance, results from the inadvertent or deliberate introduction of foreign substances into the oral mucosa. Conversely, internal pigmentation stems from natural compounds, such as hemoglobin, hemosiderin, and melanin [3]. Melanin is produced by melanocytes located in the basal layer of the epithelium and transferred to keratinocytes through melanosomes, which are organelles bound to the cell membrane. The quantity of melanin present in the body is regulated by genetic factors. However, external factors such as trauma, inflammation, hormonal fluctuations, and certain medications can induce an increase in melanin production [4]. Pigmented lesions in the oral cavity can be categorized as focal pigmentations, such as an amalgam tattoo, and multifocal pigmentations, such as physiologic pigmentation, along with pigmentations linked to systemic diseases [5]. An oral melanotic macule represents a pigmented lesion that may arise on the lips or mucous membranes, either as a focal or multifocal occurrence, resulting from heightened melanin production. It is identified by small, well-defined, brown-to-black macules [6, 7]. A biopsy is necessary to confirm the diagnosis of any pigmented lesions in the oral cavity when their causes are unclear [8]. The purpose of this article is to report an unusual case of lip melanosis resulting from fillers. This information may be beneficial in distinguishing between diagnoses and making clinical decisions.

## 2. Case Report

A 40-year-old female with a medical history of *Helicobacter pylori* infection and a gastric ulcer visited the clinic complaining of brown–black pigmentations in her upper and lower lips in November 2022. The history of the present complaint showed that the patient noticed the first spot in 2018 after a dermal filler injection, and it was a single black macule on the lower lip. After that, multiple black-pigmented macules developed on the lower and upper lips following another filler injection. She went to an oral and maxillofacial surgeon, who suggested a biopsy, but she refused. The patient is taking oral contraceptives and vitamin supplements and denied any smoking habit. She is not aware of any allergies to food or drugs. Patient consent was obtained for therapy and publication.

**Figure 1:**
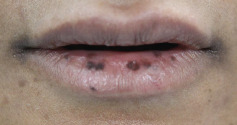
Clinical appearance of pigmentation of the lip, labial mucosa, and vermilion border.

**Figure 2:**
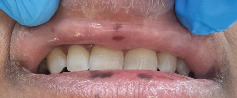
Clinical appearance of pigmentation of the lip and labial mucosa.

**Figure 3:**
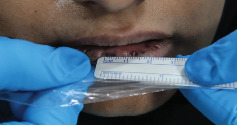
Clinical measurements of pigmentations.

**Figure 4:**
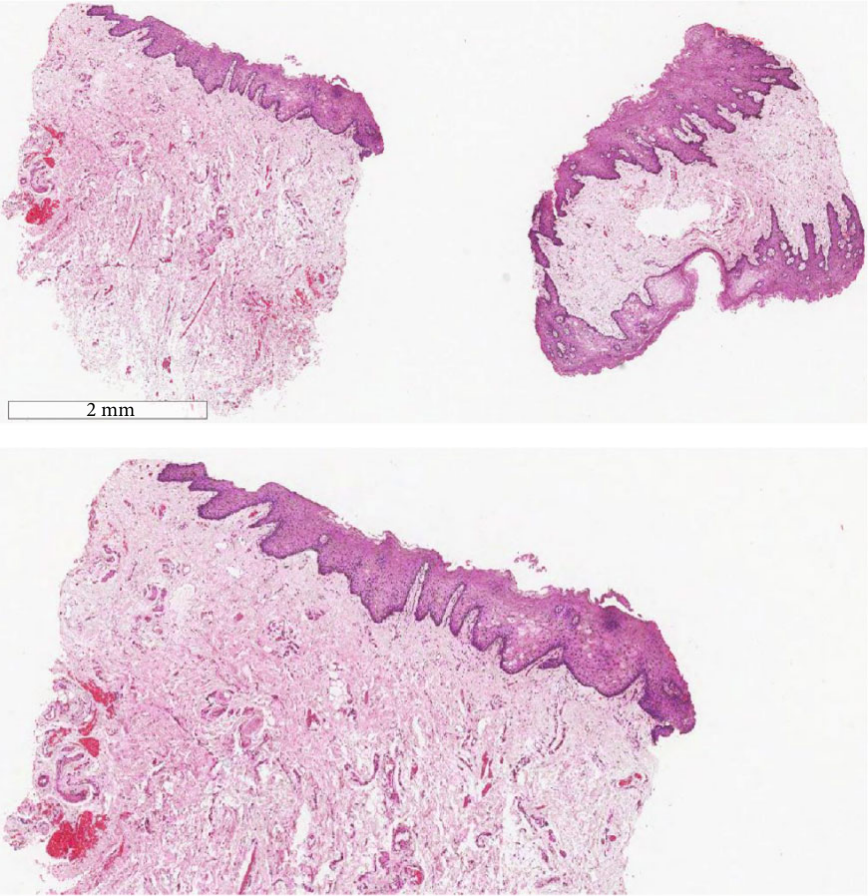
Histological H&E image showing mild acanthosis and abundant melanin in the basal layer without hyperplasia.

On examination, multiple well-demarcated, superficial, dark brown macules measuring 4∗2 mm are seen on the lips, adjacent labial mucosa, and vermilion border of the upper and lower lips (Figures 1, 2, and 3). No similar lesions were seen intra-extra orally or in the skin. The patient was convinced to undergo a biopsy and a colonoscopy. The histopathological examination of the biopsy revealed a significant presence of melanin in the basal layer without any signs of melanocytic hyperplasia or atypia. Furthermore, the colonoscopy did not reveal any abnormalities (Figure 4). A follow-up with the patient at 8 months revealed no changes in clinical appearance or complications. As a result, she was recommended for laser therapy.

## 3. Discussion

Oral pigmentation prevalence is high and therefore frequently encountered in dental practice [9]. It occurs as a result of an increase in pigments in the oral tissues, which can be attributed to various causes, endogenous and exogenous [10–12]. These causes may include physiological factors, reactive responses, neoplastic conditions, idiopathic origins, or even indications of systemic diseases [10–12]. To ensure accurate diagnosis and to exclude potentially serious conditions, such as melanoma, it is crucial to conduct a thorough assessment of the patient's medical history and a comprehensive clinical examination [13–15].

Dermal fillers are commonly used in clinical settings for cosmetic and medical purposes. Although there is a wide range of materials on the market, not all dermal fillers have been approved for cosmetic use [16, 17]. They are either temporary, semipermanent, or permanent [16, 17]. They may consist of collagen, hyaluronic acid, poly-L-lactic acid, and many other substances [16, 17]. Various early or delayed complications may arise, depending on duration and composition [16–20]. Late onset may include migration of fillers, edema, infections, nodules, or persistent change in color [18–20].

Melanocytes' activity is influenced by a variety of endogenous or exogenous factors, including endocrine, immune, inflammatory, central nervous systems, ultraviolet radiation, and medications [4, 21]. Melanogenesis is a complex and not fully understood process with multiple stages [4, 21]. Disturbances in this process can result in various pigmentation disorders, classified as hypo- or hyperpigmentation, occasionally accompanied by alterations in the melanocyte count [21].

Nonphysiological alteration of oral mucosa pigmentation can be attributed to several factors, including endocrine, chemical, or physical factors, as well as infective agents and inflammatory or neoplastic processes [11]. It has been reported that the appearance of oral melanosis in an unusual site should be followed carefully [13]. Approximately 30% of oral mucosal melanoma cases arise from areas of hyperpigmentation [4]. Examining the color, location, distribution, duration, and appearance of the pigmentation holds diagnostic importance. To establish a diagnosis, it is advantageous to assess the medical, dental, family, and social histories as well as the presence of cutaneous pigmentation or other systemic signs and symptoms. Although only a small number of lesions progress to malignancy, it remains crucial to consider the possibility of oral lesions developing into malignant tumors [15]. Biopsy is recommended in cases of unusual sites or appearance as well as aggressive behavior for a definitive diagnosis [13].

We report a case of permanent change in lip color after fillers. Dermal pigmentary changes, such as postinflammatory hyperpigmentation, are reported to be more common after ecchymosis, after superficial placement of filler material or overcorrection, and among individuals with Fitzpatrick skin Types IV–VI. Similar to Paolino et al. and Hong et al. [22, 23], histopathological examination revealed an abundance of melanin in the basal layer without melanocytic hyperplasia or atypia. To the best of our knowledge, this is the second reported case affecting the lips in the reviewed English-language literature.

## 4. Conclusion

In summary, facial and oral pigmentation could stem from a complication with fillers [22–24]. Careful history and examination are mandatory to avoid misdiagnosis. Differential diagnosis may include several etiologies, ranging from physiological to pathological conditions. Complications with fillers should be included in the clinical differential diagnosis of oral mucosa hyperpigmentation. The interaction of multiple causative agents, including technical, Tyndall effect, infection, skin, or material type, cannot be ruled out.

## Figures and Tables

**Table 1:** Classification of pigmentary disorders (adapted from Patil et al. [2]).

**Exogenous**	**Endogenous**
Melanin (brown/black or grey) • Physiological racial pigmentation • Pathological: ◦ Postinflammatory pigmentation ◦ Smokers' melanosis ◦ Melanotic neuroectodermal tumor of infancy ◦ Melanotic macule ◦ Addison's disease ◦ Peutz–Jeghers syndrome ◦ Neurofibromatosis ◦ Lentigo ◦ Nevus ◦ Melanoacanthoma ◦ Melanoma	Amalgam tattoo Heavy metal Pigmentation Drug-induced Pigmentation Other foreign body Tattoos
Hemosiderin (brown) • Ecchymosis • Petechia • Hemorrhagic mucocele • Hemochromatosis	
Hemoglobin (blue, red, and purple) • Varix • Hemangioma • Angiosarcoma • Hereditary • Hemorrhagic • Telangiectasia	

## Data Availability

Data is available.
